# Adaptive Residual Interpolation for Color and Multispectral Image Demosaicking †

**DOI:** 10.3390/s17122787

**Published:** 2017-12-01

**Authors:** Yusuke Monno, Daisuke Kiku, Masayuki Tanaka, Masatoshi Okutomi

**Affiliations:** 1 Department of Systems and Control Engineering, School of Engineering, Tokyo Institute of Technology, Meguro-ku, Tokyo 152-8550, Japan; dkiku@ok.ctrl.titech.ac.jp (D.K.); mtanaka@sc.e.titech.ac.jp (M.T.); mxo@sc.e.titech.ac.jp (M.O.); 2 Artificial Intelligence Research Center, National Institute of Advanced Industrial Science and Technology, Koto-ku, Tokyo 135-0064, Japan

**Keywords:** image sensor, Bayer color filter array, multispectral filter array, demosaicking, residual interpolation

## Abstract

Color image demosaicking for the Bayer color filter array is an essential image processing operation for acquiring high-quality color images. Recently, residual interpolation (RI)-based algorithms have demonstrated superior demosaicking performance over conventional color difference interpolation-based algorithms. In this paper, we propose adaptive residual interpolation (ARI) that improves existing RI-based algorithms by adaptively combining two RI-based algorithms and selecting a suitable iteration number at each pixel. These are performed based on a unified criterion that evaluates the validity of an RI-based algorithm. Experimental comparisons using standard color image datasets demonstrate that ARI can improve existing RI-based algorithms by more than 0.6 dB in the color peak signal-to-noise ratio and can outperform state-of-the-art algorithms based on training images. We further extend ARI for a multispectral filter array, in which more than three spectral bands are arrayed, and demonstrate that ARI can achieve state-of-the-art performance also for the task of multispectral image demosaicking.

## 1. Introduction

**Figure 1:**
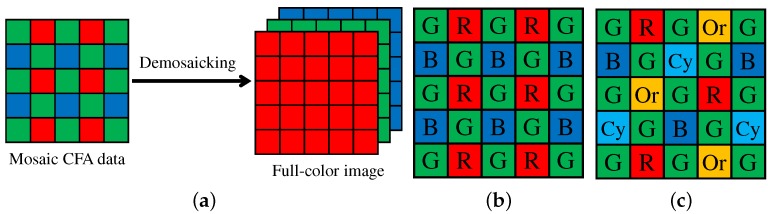
(**a**) Color image demosaicking process; (**b**) Bayer CFA [6]; (**c**) five-band MSFA [7].

A single image sensor with a color filter array (CFA) is widely used in current color digital cameras, in which only one pixel value among RGB values is recorded at each pixel [1]. The other two missing pixel values are estimated from the recorded mosaic data of RGB values by an interpolation process called demosaicking (or demosaicing) [2,3,4,5]. Figure 1a illustrates the demosaicking process, which plays a crucial role in acquiring high-quality color images using a color digital camera.

**Figure 2:**
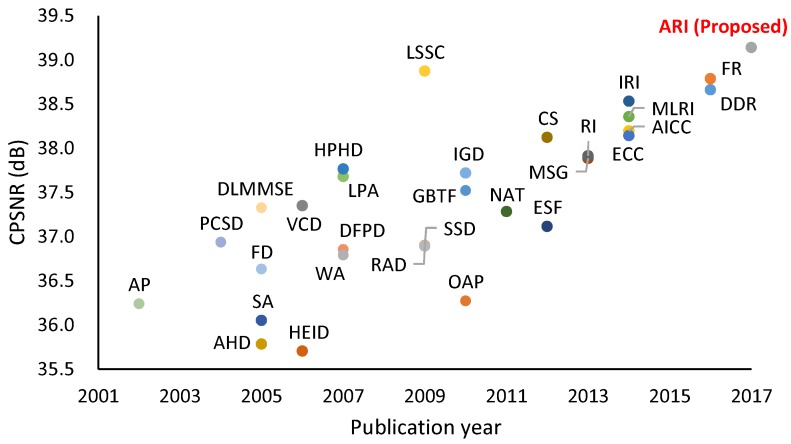
CPSNR performance of representative Bayer demosaicking algorithms on standard IMAX and Kodak 30 color images [4]. The publication year is primarily based on the journal publication except for recent algorithms presented at conferences.

The most widely used CFA is the Bayer CFA [6] (Figure 1b), for which numerous demosaicking algorithms have been proposed [2,3,4,5]. Figure 2 shows the color peak signal-to-noise ratio (CPSNR) performance of representative algorithms on a standard color image dataset [4]. The CPSNR performance has continuously been improved, suggesting the ongoing demand for more highly accurate demosaicking algorithms.

As a recent trend, residual interpolation (RI)-based algorithms have demonstrated superior demosaicking performance [8,9,10,11,12]. A key feature of the RI-based algorithms is to perform interpolation in a residual domain, where the residual is defined as the difference between a tentatively estimated pixel value and a corresponding observed pixel value. The papers [10,12] show that the residuals generally become smoother than conventional color differences, contributing to more accurate interpolation. The RI was originally proposed in [8] (denoted as original **RI** (In Section 1 and Section 2, the bold typeface is used to represent the abbreviated name of the algorithms compared in Figure 2. Hereafter, we might omit full notations of the names for the simplicity of the notations.)) and then extended in a minimized-Laplacian version (**MLRI** [9,10]) or an iterative version (**IRI** [11,12]). Recent algorithms such as **ECC** [13] and fused regression (**FR**) [14,15] also use an RI-based algorithm to assist in improving demosaicking performance. The framework of the RI-based algorithms will be reviewed in Section 3.

In this paper, we first propose adaptive residual interpolation (**ARI**) for the Bayer CFA. ARI improves the existing RI-based algorithms by introducing adaptive aspects as follows. (i) ARI adaptively combines RI and MLRI at each pixel, and (ii) ARI adaptively selects a suitable iteration number for each pixel, instead of using a common iteration number for all of the pixels, as conducted in IRI. These are performed based on a unified criterion that evaluates the validity of an RI-based algorithm. Figure 2 demonstrates that ARI can improve the existing RI-based algorithms by more than 0.6 dB in CPSNR and can outperform state-of-the-art algorithms based on training images, such as **LSSC** [16] and **FR** [14,15].

We next consider the task of multispectral image demosaicking. The CFA and demosaicking technologies are extensible for multispectral imaging with a single image sensor [17]. The only modification required in hardware is to replace the CFA with a multispectral filter array (MSFA), in which more than three spectral bands are arrayed. Figure 1c shows a representative five-band MSFA, consisting of typical RGB bands and additional orange and cyan bands (denoted as Or and Cy bands, respectively) in the visible spectrum [7]. The multispectral extension of the CFA has attracted increasing attention because of its potential for compact and low-cost multispectral image acquisition. However, the demosaicking process for an MSFA poses a challenging problem owing to the very sparse sampling of each spectral band in the MSFA. To address this challenge, we extend our proposed ARI for multispectral image demosaicking, considering the five-band MSFA of Figure 1c. Experimental comparisons using several multispectral image datasets demonstrate that ARI can achieve state-of-the-art performance also for the task of multispectral image demosaicking.

An earlier version of this paper was published in [18]. This paper provides three major extensions. First, we improve the demosaicking accuracy of the R and B bands in color image demosaicking by incorporating ARI into not only the G band interpolation (as performed in [18]), but also the R and B bands’ interpolation. Second, we extend ARI for multispectral image demosaicking and demonstrate that ARI can achieve state-of-the-art performance. Third, we include the detailed explanation of our algorithm and extensive experimental comparisons with existing algorithms.

The rest of this paper is organized as follows. Section 2 briefly reviews existing Bayer and multispectral demosaicking algorithms. Section 3 outlines the framework of RI-based algorithms. Section 4 explains our proposed ARI for the Bayer CFA. Section 5 extends ARI for multispectral image demosaicking. Section 6 presents experimental results, and Section 7 concludes the paper.

## 2. Related Works

### 2.1. Bayer Demosaicking Algorithms

Numerous demosaicking algorithms have been proposed for the Bayer CFA. While referring to the well-categorized review in [19], we classify existing algorithms into several categories.

Interpolation-based algorithms first interpolate the G band, which has a sampling density double that of the R and B bands. Then, the R and B bands are interpolated in a color ratio [20,21,22] or a color difference domain [23,24] based on the assumption that the color ratios (i.e., R/G and B/G) or the color differences (i.e., R-G and B-G) are smooth within a local area of an image. In practice, color differences are used more often than color ratios because of their stable properties [25].

The interpolation-based algorithms mainly focus on improving the interpolation accuracy of the G band using directional interpolation that combines two interpolation results along the horizontal and vertical directions. The two results are combined (or one result is selected) effectively based on such a primary-consistent soft-decision (**PCSD** [26]), homogeneity metrics on the CIE Lab color space (**AHD** [27]), directional linear minimum mean square-error estimation (**DLMMSE** [28]), variance of color differences (**VCD** [29]), directional filtering and a posteriori decision (**DFPD** [30]), heterogeneity-projection hard-decision (**HPHD** [31]), local polynomial approximation (**LPA** [32]), integrated gradients (**IGD** [33]), gradients of color differences (**GBTF** [34]) and multi-scale color gradients (**MSG** [35]). Some algorithms update interpolated pixel values once or iteratively (**SA** [25], **HEID** [36] and **ESF** [37]). Non-local self-similarities (**SSD** [38,39], **NAT** [40] and **AICC** [41,42]) or more than two directions (**CS** [43]) are also used. The algorithm (**ECC** [13]) effectively combines band-independent and color difference interpolation results.

RI-based algorithms have shown superior demosaicking performance in recent years. The RI-based algorithms also interpolate the G band first. Then, they generate tentative estimates of the R and B bands (denoted as $\overset{ˇ}{R}$ and $\overset{ˇ}{B}$, respectively) from the interpolated G band. Then, the residuals (denoted as R-$\overset{ˇ}{R}$ and B-$\overset{ˇ}{B}$, respectively) are calculated and interpolated. The RI-based algorithms are motivated by the observation that the residuals generally become smoother than the conventional color differences (i.e., R-G and B-G), contributing to more accurate interpolation. After the original RI was proposed (**RI** [8]), several extensions were introduced using minimized-Laplacian (**MLRI** [9,10]), iteration (**IRI** [11,12]) or four directionality [44]. The RI-based algorithms will be further reviewed in Section 3.

Frequency-domain algorithms first transform the mosaic CFA image into the frequency domain, where the CFA image is expressed as a combination of luminance and chrominance components. These components are then separated by frequency filtering and finally converted into the RGB components (**FD** [45,46]). In this category, researchers focus on designing effective frequency filtering algorithms such as adaptive filtering [47] and least-squares luma-chroma demultiplexing [48] (The source code of this algorithm is publicly available. However, we excluded this algorithm from comparison in Figure 2 because the necessary trained filters are provided only for the Kodak dataset.).

Wavelet-based algorithms perform a sub-band analysis of the RGB or luminance image. The alternative projection algorithm (**AP** [49]) decomposes initially interpolated R, G and B images into sub-bands and iteratively updates the high-frequency sub-bands of the R and B images in accordance with those of the G image. This algorithm is accelerated using one-step implementation without iteration (**OAP** [50]). In another algorithm, a wavelet analysis of the luminance component is performed to estimate interpolation weights for the horizontal and vertical directions (**WA** [51]).

Reconstruction-based algorithms solve the demosaicking process as an optimization problem with a proper regularization or prior. A regularization approach (**RAD** [52]) uses prior knowledge regarding natural color images, such as smoothness and inter-band correlations. Other algorithms are based on dictionary learning with non-local sparse models (**LSSC** [16]) or the theory of compressed sensing [53].

Regression-based algorithms learn efficient regressors based on training images [14,15]. Directional difference regression (**DDR** [15]) learns the regressors that estimate directional color differences of the training images (as ground truths without mosaicking) closest to those calculated from the input mosaic CFA data. To improve performance, fused regression (**FR** [15]) fuses the DDR and the other regressors that estimate the RGB values of the training images (as ground truths without mosaicking) closest to the RGB values initially interpolated using MLRI [9,10].

Short summary: Generally, demosaicking algorithms based on training images, such as **LSSC** [16], **DDR** [15] and **FR** [15], can offer high performance results, as demonstrated in Figure 2. In contrast, interpolation-based algorithms are flexibly applicable to any kind of input data without relying on training images. This property is important in fields such as medical and multispectral imaging, because obtaining high-quality and sufficient training images is laborious. In this paper, we focus on improving the RI-based algorithms as will be explained in Section 4. We refer to the survey papers [2,3,4,5] for complementary information.

### 2.2. Multispectral Demosaicking Algorithms

While the study of Bayer demosaicking algorithms has a long history, that of multispectral demosaicking algorithms has attracted attention only in recent years. Because there is no wide-spread MSFA currently, research on multispectral demosaicking algorithms has been conducted in conjunction with the design of an MSFA.

Miao et al. proposed a generic algorithm for designing an MSFA with an arbitrary number of spectral bands [54]. They also proposed a general binary tree-based edge-sensing (BTES) demosaicking algorithm [55] for the MSFA designed by the generic method. The BTES algorithm recursively performs edge-sensing interpolation based on a binary tree. Although the generic and the BTES algorithms are useful in providing a general framework, the performance of classical edge-sensing interpolation is limited.

Monno et al. proposed a five-band MSFA [7], as shown in Figure 1c. This five-band MSFA consists of typical RGB bands and additional Or and Cy bands in the visible spectrum. The advantage of this MSFA lies in keeping the sampling density of the G band in the MSFA as high as that in the Bayer CFA. Based on that advantage, several efficient demosaicking algorithms have been proposed [7,56,57,58,59,60]. These algorithms first interpolate the most densely-sampled G band, which is effectively used as a guide for interpolating the other bands. In [56], adaptive kernel upsampling (AKU) was proposed to generate an interpolated five-band image using the interpolated G band as a guide for estimating interpolation directions of the other bands. In [7,57], guided filtering (GF) [61] is applied to obtain an interpolated result. In [58], an RI-based algorithm was incorporated into the interpolation of both the G band and the other bands. In [59,60], the high-frequency component of the G band was effectively exploited for interpolating the other bands based on the inter-band correlation analysis.

Recently, multispectral demosaicking algorithms for other types of MSFA have also been proposed, such as the algorithm based on linear minimum mean square errors for an eight-band MSFA [62] and the algorithm using a pseudo-panchromatic image for a 16-band MSFA [63]. We refer to the paper [17] for a comprehensive review, in which other MSFAs, such as uniform MSFAs [64,65] and a seven-band MSFA [66], and demosaicking algorithms for those MSFAs are introduced.

In this paper, we propose a multispectral demosaicking algorithm for the five-band MSFA of Figure 1c, as will be described in Section 5. We consider that the use of that MSFA is a reasonable choice for three reasons. (i) As reported in [7], the considered MSFA has demonstrated better performance in comparison with other five-band MSFAs generated by the generic algorithm. (ii) The considered MSFA has already been realized in hardware as a prototype sensor [7]. (iii) Jia et al. have also used the same MSFA arrangement and have reported its effectiveness by the frequency domain analysis of the MSFA [67,68]. These facts suggest the potential of the considered MSFA.

## 3. Residual Interpolation Framework

In this section, we review the RI framework and three specific RI-based algorithms: RI [8], MLRI [9,10] and IRI [11,12].

### 3.1. General Processing Flow

**Figure 3:**
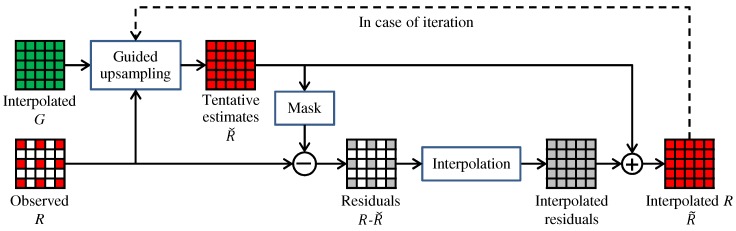
The RI framework.

Figure 3 outlines the RI framework. Here, we assume that the G band interpolation is already completed and take the R band interpolation as an example to explain the framework. The RI framework consists of four steps. (i) Tentative estimates of the R band (denoted as $\overset{ˇ}{R}$) are generated from the interpolated G band by guided upsampling of the observed R values. (ii) Residuals (denoted as *R*-$\overset{ˇ}{R}$) are calculated by taking the differences between the tentative estimates and the observed R values. (iii) The residuals are interpolated. (iv) The tentative estimates are added to the interpolated residuals to obtain the interpolated R band (denoted as $\tilde{R}$). The key effect of the RI framework is that the residuals become smoother than the conventional color differences (i.e., *R*-*G*) by effectively generating the tentative estimates [10,12]. This property increases the interpolation accuracy. A similar procedure can be applied for the G band interpolation, as will be detailed in Section 4. In the following, we explain three RI-based algorithms, which are different regarding tentative estimates generation.

### 3.2. Original RI

Original RI [8] generates the tentative estimates using GF [61]. For each local image window, GF generates the tentative estimates as a linear transformation of a guide. Here, the interpolated G band is used as the guide. The tentative estimates in a local window $\omega_{p,q}$ around a pixel (p,q) are expressed as:

(1) $$\begin{array}{c} \begin{array}{c} {\overset{ˇ}{R}}_{i,j}=a_{p,q}G_{i,j}+b_{p,q}\;,\;\;\;\forall_{i,j}\in \omega_{p,q}\;, \end{array} \end{array}$$

where ${\overset{ˇ}{R}}_{i,j}$ represents the tentative estimate at a pixel (i,j) in the window and $\left(a_{p,q},b_{p,q}\right)$ is the pair of linear coefficients assumed to be constant in the window.

The cost function for $\left(a_{p,q},b_{p,q}\right)$ to be minimized is expressed as:

(2) $$\begin{array}{cc} E\left(a_{p,q},b_{p,q}\right) & =\sum_{i,j\in \omega_{p,q}}{\left(M_{i,j}\left(R_{i,j}-{\overset{ˇ}{R}}_{i,j}\right)\right)}^{2},\\ & =\sum_{i,j\in \omega_{p,q}}{\left(M_{i,j}\left(R_{i,j}-a_{p,q}G_{i,j}-b_{p,q}\right)\right)}^{2}, \end{array}$$

where $M_{i,j}$ is a binary mask at the pixel (i,j) that takes the value one for the observed R pixels and zero for the others (Original GF [61] has a smoothness term ϵa. For our purpose, the smoothness parameter ϵ is set as a very small value just to avoid division by zero, and thus, we omit the smoothness term from the cost function in Equations (2) and (3).). The above cost function indicates that the original RI minimizes the residuals (i.e., *R*-$\overset{ˇ}{R}$) themselves. Since the linear coefficients are calculated in a sliding window, the overlaps of the windows are averaged uniformly or with weights [10].

### 3.3. Minimized-Laplacian RI

MLRI [9,10] generates the tentative estimates by minimizing the Laplacian energy of the residuals, instead of the residuals themselves. For this purpose, GF is modified as follows. For each local window, the cost function for the gain component $a_{p,q}$ is expressed as:

(3) $$\begin{array}{cc} E\left(a_{p,q}\right) & =\sum_{i,j\in \omega_{p,q}}{\left(M_{i,j}{\tilde{\nabla }}^{2}\left(R_{i,j}-{\overset{ˇ}{R}}_{i,j}\right)\right)}^{2},\\ & =\sum_{i,j\in \omega_{p,q}}{\left(M_{i,j}{\tilde{\nabla }}^{2}\left(R_{i,j}-a_{p,q}G_{i,j}-b_{p,q}\right)\right)}^{2},\\ & =\sum_{i,j\in \omega_{p,q}}{\left({\tilde{\nabla }}^{2}\left(R_{i,j}^{M}\right)-a_{p,q}{\tilde{\nabla }}^{2}\left(G_{i,j}^{M}\right)\right)}^{2}, \end{array}$$

where ${\tilde{\nabla }}^{2}\left(\cdot \right)$ represents an approximate Laplacian value, and $R_{i,j}^{M}$ and $G_{i,j}^{M}$ are the R and the G values masked by $M_{i,j}$, respectively. The approximate Laplacian value is calculated from the masked mosaic data by convolving the following sparse Laplacian filter:

(4) $$\begin{array}{c} \left[\begin{array}{ccccc} 0 & 0 & -1 & 0 & 0\\ 0 & 0 & 0 & 0 & 0\\ -1 & 0 & 4 & 0 & -1\\ 0 & 0 & 0 & 0 & 0\\ 0 & 0 & -1 & 0 & 0 \end{array}\right]. \end{array}$$

Although the bias component $b_{p,q}$ does not affect the minimization of the Laplacian energy (i.e., ${\tilde{\nabla }}^{2}\left(b_{p,q}\right)=0$), $b_{p,q}$ is determined by minimizing Equation (2) under a given $a_{p,q}$.

### 3.4. Iterative RI

IRI [11,12] introduces an iterative manner to the RI framework. The iterative manner is indicated by the dashed line in Figure 3, where the interpolated R band is used as the guide in the next iteration. In [11], the iteration is stopped based on a criterion that is defined by the magnitude and the smoothness of the residuals at the *k*-th iteration (i.e., *R*-${\overset{ˇ}{R}}_{k}$) to assess how effectively the tentative estimates fit the observed values. In [12], a different stopping criterion is used to assess whether the new interpolation result becomes sufficiently close to the previous iteration result with a proper threshold (i.e., $|{\tilde{R}}_{k}-{\tilde{R}}_{k-1}|<\gamma$). Both in [11,12], the iteration is stopped in a global manner, which means that a common iteration number is used for all pixels based on the criterion combined for all pixels.

## 4. Proposed Bayer Demosaicking Algorithm

### 4.1. Interpolation of the G Band

**Figure 4:**
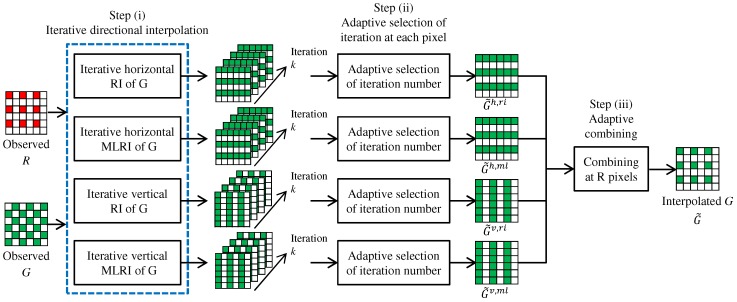
Overall flow of the G interpolation at R pixels using ARI.

Our proposed algorithm first interpolates the G band. The overall flow of the G interpolation is illustrated in Figure 4. Here, we only explain the G interpolation for the R pixels. The G interpolation for the B pixels is performed in the same manner.

Our proposed G interpolation algorithm consists of three steps. (i) The G interpolation at the R lines, which represent the pixel rows that contain the R pixels, is performed in the horizontal and vertical directions. For each direction, RI and MLRI are applied in an iterative manner, respectively. As a result of each directional interpolation, a set of directionally-interpolated G results, where each result corresponds to one iteration, is generated. (ii) For each directional interpolation, a suitable iteration number is selected adaptively at each pixel from the set of directionally-interpolated G results. (iii) All directional results are adaptively combined by a weighted averaging at each R pixel to obtain the final G interpolation result. The sequence of the above steps is called ARI in the sense that it adaptively combines RI and MLRI and selects the iteration number at each pixel. Each step is detailed as below.

**Figure 5:**
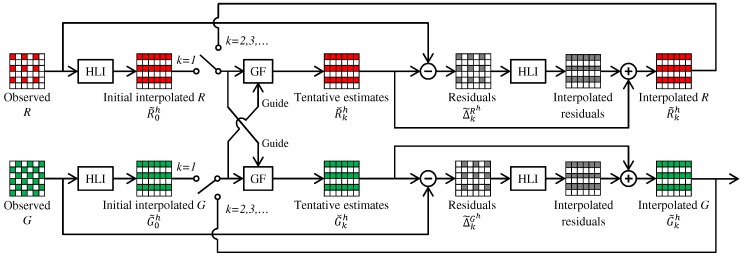
Flow of the iterative horizontal G interpolation at the R lines by RI or MLRI.

Step (i): Iterative directional interpolation. In this step, RI and MLRI are applied for iterative directional interpolation. Figure 5 illustrates the flow of the G interpolation at the R lines in the horizontal direction. The G interpolation in the vertical direction is performed in the same manner.

First, horizontal linear interpolation (HLI) of the R and the G bands is performed at the R lines to obtain initial interpolation results as:

(5) $$\begin{array}{c} \begin{array}{cc} {\tilde{R}}_{(i,j),0}^{h} & =(R_{\left(i-1,j\right)}+R_{\left(i+1,j\right)})/2\;,\;\;\mathrm{at}\;a\;G\;\mathrm{pixel}\;\mathrm{in}\;\mathrm{an}\;R\;\mathrm{line},\\ {\tilde{G}}_{(i,j),0}^{h} & =(G_{\left(i-1,j\right)}+G_{\left(i+1,j\right)})/2\;,\;\;\mathrm{at}\;\mathrm{an}\;R\;\mathrm{pixel}, \end{array} \end{array}$$

where $\tilde{R}$ and $\tilde{G}$ represent the interpolated pixel values, the subscript (i,j),0 indicates the initial interpolation result at a pixel (i,j) and the superscript *h* represents the horizontal direction.

Then, at each *k*-th iteration, tentative estimates of R and G are generated by linear transformations of the previous interpolation results (if k=1, the initial interpolation results are used) as:

(6) $$\begin{array}{c} \begin{array}{cc} {\overset{ˇ}{R}}_{(i,j),k}^{h} & =a_{(i,j),k}^{r}{\tilde{G}}_{(i,j),k-1}^{h}+b_{(i,j),k}^{r}\;,\\ {\overset{ˇ}{G}}_{(i,j),k}^{h} & =a_{(i,j),k}^{g}{\tilde{R}}_{(i,j),k-1}^{h}+b_{(i,j),k}^{g}\;, \end{array} \end{array}$$

where $\overset{ˇ}{R}$ and $\overset{ˇ}{G}$ represent the tentative estimates and $\left(a^{r},b^{r}\right)$ and $\left(a^{g},b^{g}\right)$ are the linear coefficients. As described in Section 3.2 and Section 3.3, RI and MLRI calculate the linear coefficients using GF [61] and its modified version, respectively. Here, $\tilde{G}$ is used as the guide for generating $\overset{ˇ}{R}$ and vice versa. At each local window, RI and MLRI calculate the linear coefficients by minimizing the following costs, respectively.

(7) $$\begin{array}{c} e_{(i,j),k}^{R^{h}}=\left\{\begin{array}{cc} {\overset{ˇ}{R}}_{(i,j),k}^{h}-{\tilde{R}}_{(i,j),k-1}^{h}\;, & \mathrm{in}\;\mathrm{RI},\;\\ {\tilde{\nabla }}^{2}\left({\overset{ˇ}{R}}_{(i,j),k}^{h}\right)-{\tilde{\nabla }}^{2}\left({\tilde{R}}_{(i,j),k-1}^{h}\right)\;, & \mathrm{in}\;\mathrm{MLRI}, \end{array}\right. \end{array}$$

(8) $$\begin{array}{c} e_{(i,j),k}^{G^{h}}=\left\{\begin{array}{cc} {\overset{ˇ}{G}}_{(i,j),k}^{h}-{\tilde{G}}_{(i,j),k-1}^{h}\;, & \mathrm{in}\;\mathrm{RI},\;\\ {\tilde{\nabla }}^{2}\left({\overset{ˇ}{G}}_{(i,j),k}^{h}\right)-{\tilde{\nabla }}^{2}\left({\tilde{G}}_{(i,j),k-1}^{h}\right)\;, & \mathrm{in}\;\mathrm{MLRI}, \end{array}\right. \end{array}$$

where $e^{R^{h}}$ and $e^{G^{h}}$ represent the costs for the tentative estimates of R and G, respectively.

After generating the tentative estimates, the residuals are calculated as:

(9) $$\begin{array}{c} \begin{array}{cc} {\tilde{\Delta }}_{(i,j),k}^{R^{h}} & =R_{\left(i,j\right)}-{\overset{ˇ}{R}}_{(i,j),k}^{h}\;,\;\;\mathrm{at}\;\mathrm{an}\;R\;\mathrm{pixel},\\ {\tilde{\Delta }}_{(i,j),k}^{G^{h}} & =G_{\left(i,j\right)}-{\overset{ˇ}{G}}_{(i,j),k}^{h}\;,\;\;\mathrm{at}\;a\;G\;\mathrm{pixel}\;\mathrm{in}\;\mathrm{an}\;R\;\mathrm{line}. \end{array} \end{array}$$

Then, the residuals are interpolated by the HLI as:

(10) $$\begin{array}{c} \begin{array}{cc} {\tilde{\Delta }}_{(i,j),k}^{R^{h}} & =({\tilde{\Delta }}_{(i-1,j),k}^{R^{h}}+{\tilde{\Delta }}_{(i+1,j),k}^{R^{h}})/2\;,\;\;\mathrm{at}\;a\;G\;\mathrm{pixel}\;\mathrm{in}\;\mathrm{an}\;R\;\mathrm{line},\\ {\tilde{\Delta }}_{(i,j),k}^{G^{h}} & =({\tilde{\Delta }}_{(i-1,j),k}^{G^{h}}+{\tilde{\Delta }}_{(i+1,j),k}^{G^{h}})/2\;,\;\;\mathrm{at}\;\mathrm{an}\;R\;\mathrm{pixel}. \end{array} \end{array}$$

Finally, the tentative estimates are added to obtain the *k*-th horizontally interpolated results as:

(11) $$\begin{array}{c} \begin{array}{c} {\tilde{R}}_{(i,j),k}^{h}={\tilde{\Delta }}_{(i,j),k}^{R^{h}}+{\overset{ˇ}{R}}_{(i,j),k}^{h}\;,\\ {\tilde{G}}_{(i,j),k}^{h}={\tilde{\Delta }}_{(i,j),k}^{G^{h}}+{\overset{ˇ}{G}}_{(i,j),k}^{h}\;. \end{array} \end{array}$$

In the iterative manner [11,12], the tentative estimates are updated in accordance with Equation (6) using the obtained interpolation results. In [11,12], the iteration is stopped globally based on the criterion described in Section 3.4, meaning that a common iteration number is used for all of the pixels. In contrast, ARI generates a set of directionally-interpolated results, in which each result corresponds to one iteration, and adaptively selects a suitable iteration number for each pixel in the next step.

Step (ii): Adaptive selection of iteration number. In this step, for each directional interpolation, a suitable iteration number is adaptively selected at each pixel. This is performed based on a criterion similar to that in [11]. Here, we only explain the criterion for the horizontal direction. The criterion for the vertical direction is calculated in the same manner.

We define the criterion in a pixel-by-pixel manner, instead of the global manner in [11], based on the following differences.

(12) $$\begin{array}{c} \begin{array}{c} d_{(i,j),k}^{R^{h}}={\overset{ˇ}{R}}_{(i,j),k}^{h}-{\tilde{R}}_{(i,j),k-1}^{h}\;,\\ d_{(i,j),k}^{G^{h}}={\overset{ˇ}{G}}_{(i,j),k}^{h}-{\tilde{G}}_{(i,j),k-1}^{h}\;, \end{array} \end{array}$$

where $d^{R^{h}}$ and $d^{G^{h}}$ represent the differences between the *k*-th tentative estimates and the previous interpolation results. These differences assess how effectively the tentative estimates converge at the *k*-th iteration. The criterion value for a pixel (i,j) at the *k*-th iteration is defined based on the magnitude and the smoothness of the above differences as:

(13) $$\begin{array}{c} \begin{array}{c} c_{(i,j),k}^{h}={\left(d_{(i,j),k}^{h}\right)}^{m}\cdot {\left(\delta d_{(i,j),k}^{h}\right)}^{n}\;, \end{array} \end{array}$$

where $d_{(i,j),k}^{h}=|d_{(i,j),k}^{R^{h}}\left|+\right|d_{(i,j),k}^{G^{h}}|$ and $\delta d_{(i,j),k}^{h}=|d_{(i-1,j),k}^{R^{h}}-d_{(i+1,j),k}^{R^{h}}\left|+\right|d_{(i-1,j),k}^{G^{h}}-d_{(i+1,j),k}^{G^{h}}|$. The above criterion value becomes small if the magnitude of the differences (minimized in RI) or the smoothness of the differences (minimized in MLRI) is small. The parameters (m,n) are set empirically as (2,1), the same parameter values as used in [11].

Based on criterion values corresponding to each pixel, the suitable iteration number $k_{best}$ is adaptively selected at each pixel as:

(14) $$\begin{array}{c} \begin{array}{c} k_{best}=\arg\min_{k}g\left(c_{(i,j),k}^{h}\right)\;, \end{array} \end{array}$$

where the function g(·) represents spatial Gaussian smoothing. We empirically chose σ=2 for the spatial Gaussian smoothing of criterion values and k=11 for the maximum iteration number. Although our iteration strategy does not guarantee theoretical convergence at each pixel, the selection of the most suitable iteration number based on the minimal criterion value performs well. Hereafter, we remove the subscript *k*, indicating that the suitable iteration number $k_{best}$ has already been selected at each pixel.

Step (iii): Adaptive combining of all directional results. In this step, directional interpolation results of RI and MLRI are combined by the weighted averaging as:

(15) $$\begin{array}{c} \begin{array}{c} {\tilde{G}}_{\left(i,j\right)}=\frac{w_{\left(i,j\right)}^{h,ri}{\tilde{G}}_{\left(i,j\right)}^{h,ri}+w_{\left(i,j\right)}^{h,ml}{\tilde{G}}_{\left(i,j\right)}^{h,ml}+w_{\left(i,j\right)}^{v,ri}{\tilde{G}}_{\left(i,j\right)}^{v,ri}+w_{\left(i,j\right)}^{v,ml}{\tilde{G}}_{\left(i,j\right)}^{v,ml}}{w_{\left(i,j\right)}^{h,ri}+w_{\left(i,j\right)}^{h,ml}+w_{\left(i,j\right)}^{v,ri}+w_{\left(i,j\right)}^{v,ml}}, \end{array} \end{array}$$

where *h* and *v* represent the horizontal and the vertical directions and ri and ml represent the results of RI and MLRI, respectively. Each weight is calculated based on the smoothed criterion value as:

(16) $$\begin{array}{c} \begin{array}{c} w_{\left(i,j\right)}^{h,ri}=1/g\left(c_{\left(i,j\right)}^{h,ri}\right)\;,\;\;\;\;w_{\left(i,j\right)}^{h,ml}=1/g\left(c_{\left(i,j\right)}^{h,ml}\right)\;,\\ w_{\left(i,j\right)}^{v,ri}=1/g\left(c_{\left(i,j\right)}^{v,ri}\right)\;,\;\;\;\;w_{\left(i,j\right)}^{v,ml}=1/g\left(c_{\left(i,j\right)}^{v,ml}\right)\;, \end{array} \end{array}$$

where a small criterion value contributes to a large weight.

### 4.2. Interpolation of the R and B Bands

In IRI [11,12], iteration is performed only for the G interpolation, while the R and B interpolations are performed without iteration. In contrast, we fully incorporate ARI not only into the G interpolation, but also into the R and B interpolations.

**Figure 6:**
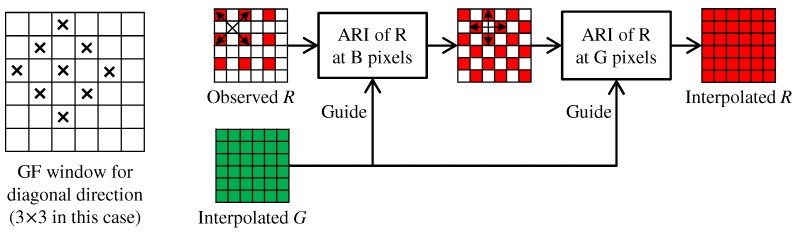
Flow of the R interpolation for the Bayer CFA. A window of GF for the diagonal direction is shown in the left.

Figure 6 shows the flow of the R interpolation. We take a progressive approach [55] as follows. (i) The R values at the B pixels are interpolated using ARI along the diagonal directions. (ii) The R values at the G pixels are interpolated using ARI along the horizontal and vertical directions. In ARI, the interpolated G band is fixed throughout the process and is used as the guide for generating the tentative estimates of the R band at each iteration. The B interpolation is performed in the same manner. In our experiments, a maximum of two iterations provides sufficiently high performance results for the R and B interpolations. This iteration process improves the demosaicking performance by approximately 0.13 dB in PSNR of the R and B bands and 0.1 dB in CPSNR.

### 4.3. Window Size of GF

In our implementation, we empirically set the window size of GF in Equation (6) as follows. Here, we explain the window size for the horizontal interpolation. The window size for the vertical interpolation is set symmetrically.

In the G interpolation, the window size of GF (denoted as height (*H*) × width (*W*)) is initially set as 3×5 for horizontal RI and 1×9 for horizontal MLRI. As conducted in [11], the window size is gradually enlarged at each *k*-th iteration as $H_{k}=H_{k-1}+2$ and $W_{k}=W_{k-1}+2$. In the first step of the R and B interpolations, the GF window is set as shown in the left of Figure 6. We initially use the diagonal 5×5 window for diagonal RI and the diagonal 1×5 window for diagonal MLRI. The window size is then diagonally enlarged in the same manner as the horizontal G interpolation. In the second step of the R and B interpolations, the window size of GF is initially set as 5×5 for horizontal RI and 1×5 for horizontal MLRI. The window size is then enlarged as $H_{k}=H_{k-1}+2$ and $W_{k}=W_{k-1}+2$. The window enlargement greatly affects the demosaicking performance and improves the CPSNR by more than 1 dB compared with the case without the enlargement.

## 5. Multispectral Extension

In this section, we extend our proposed ARI for multispectral image demosaicking with the five-band MSFA of Figure 1c. Our proposed multispectral demosaicking algorithm using ARI first interpolates the G band, which is the most densely sampled in the five-band MSFA. Then, the other four bands are interpolated using the G band as a guide.

**Figure 7:**
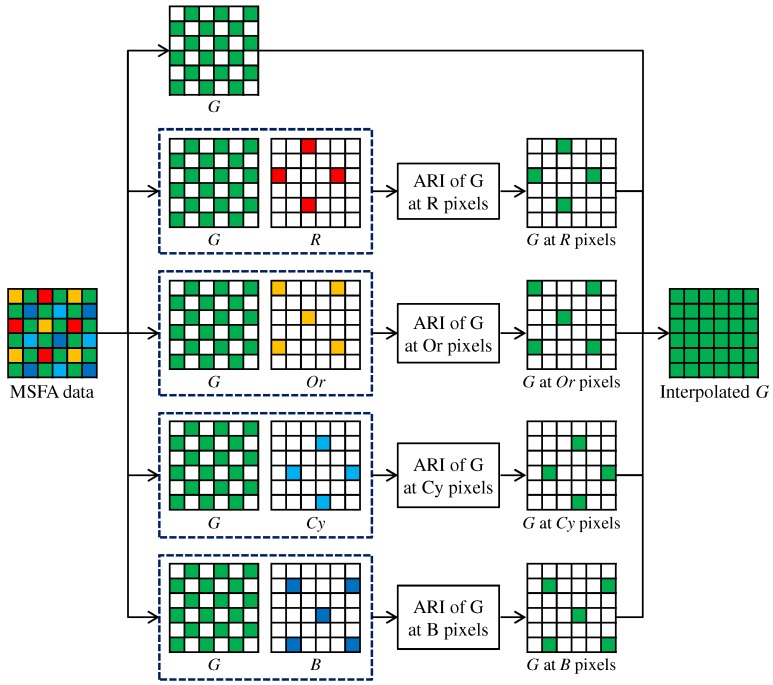
Flow of the G interpolation for the five-band MSFA.

**Figure 8:**
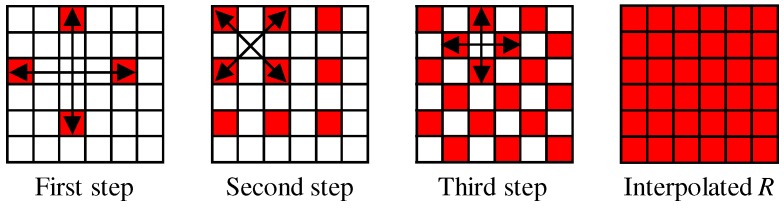
Three steps of the R interpolation for the five-band MSFA.

Figure 7 shows the flow of the G interpolation for the five-band MSFA. The G interpolation is decomposed into four streams, which correspond to the G interpolation at the R, Or, Cy and B pixels, respectively. In each stream, the G interpolation is performed in the same manner as that for the Bayer CFA (Figure 4 and Figure 5), considering the differences in the sampling patterns. A notable difference is that the linear interpolation is performed using the filter [1/4,1/2,3/4,1,3/4,1/2,1/4] for the horizontal direction and the filter ${\left[1/4,1/2,3/4,1,3/4,1/2,1/4\right]}^{T}$ for the vertical direction. The interpolation of the other four bands is performed in the progressive approach, as explained in Section 4.2. For the five-band MSFA, three steps of Figure 8, in which the R interpolation is taken as an example, are performed.

## 6. Experimental Results

### 6.1. Performance of Bayer Demosaicking Algorithms

To evaluate the performance of Bayer demosaicking algorithms, we used two standard color image datasets, the IMAX and the Kodak datasets. These two datasets are used for the benchmark comparison in the representative survey paper [4]. The IMAX dataset contains 18 images of size 500 × 500 pixels [40]. The Kodak dataset contains 12 images of size 768 × 512 pixels [4].

Our proposed ARI (Code available at http://www.ok.sc.e.titech.ac.jp/res/DM/RI.html.) was compared extensively with 29 algorithms that were briefly reviewed in Section 2. The source codes are publicly available or executable at their authors’ websites. Only for IRI [11,12], we asked the authors to send us the resulting images, because the source code is not publicly available. We used a default set of parameters provided by the authors for the evaluation. DDR [15] and FR [15] have two sets of parameters, in which each set is optimized for each dataset. To evaluate all algorithms under a common parameter condition in both datasets, we selected a better set of parameters for DDR and FR in terms of the average CPSNR performance on both datasets. We also included the previous version of ARI (denoted as ARI (Previous)) [18] as a reference. We omit ten border pixels from the evaluation to discount implementation errors in those pixels.

Table 1 summarizes the average PSNR and CPSNR performance. For the IMAX dataset, our proposed ARI outperforms all existing algorithms in the CPSNR performance. The regression-based algorithms based on training images, i.e., DDR and FR, follow ARI. The existing RI-based algorithms such as MLRI and IRI, also offer reasonably high performance results.

For the Kodak dataset, the algorithm based on dictionary learning, i.e., LSSC [16], offers the best performance. The interpolation-based algorithm with multiscale gradients, i.e., MSG [35], follows LSSC. For the average of all images for both datasets, our proposed ARI improves IRI by approximately 0.6 dB in CPSNR and outperforms all existing algorithms, including state-of-the-art algorithms based on training images, such as LSSC and FR. Let us note that FR uses MLRI as a source of initial interpolation to learn efficient regressors. Therefore, the integration of our proposed ARI into FR has the potential to further improve the performance. Table 2 summarizes the average structural similarity (SSIM) [69] performance, which demonstrates results similar to those of the PSNR performance in Table 1.

**Figure 9:**
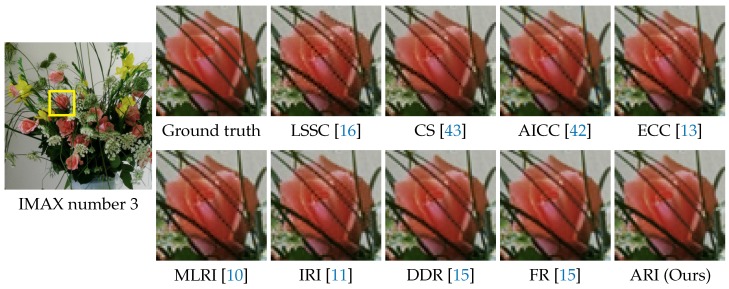
Visual comparison of the demosaicking results on the IMAX number 3 image. The results of the top nine algorithms for the average CPSNR in Table 1 are shown.

**Figure 10:**
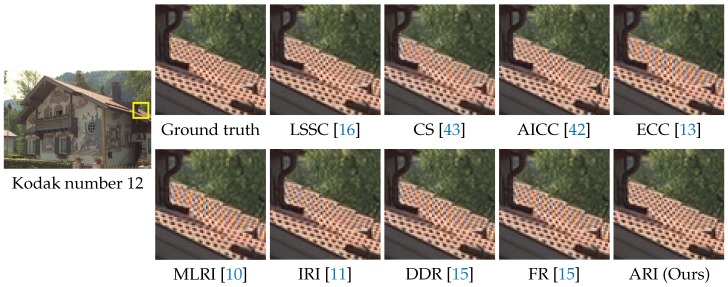
Visual comparison of the demosaicking results on the Kodak number 12 image. The results of the top nine algorithms for the average CPSNR in Table 1 are shown.

Figure 9 shows the visual comparison of demosaicking results on the IMAX number 3 image. In the figure, the results of the top nine algorithms, which offer average CPNR performance greater than 38 dB in Table 1, are shown. One can see that ARI can generate a high quality image without severe zipper artifacts. FR also can generate a comparable result. Figure 10 shows the visual comparison of demosaicking results on the Kodak number 12 image. One can see that ARI can reduce severe color artifacts that appear in the results of existing algorithms other than LSSC. Both the numerical and visual comparisons validate that our proposed ARI can achieve state-of-the-art performance for the color image demosaicking with the Bayer CFA.

**Figure 11:**
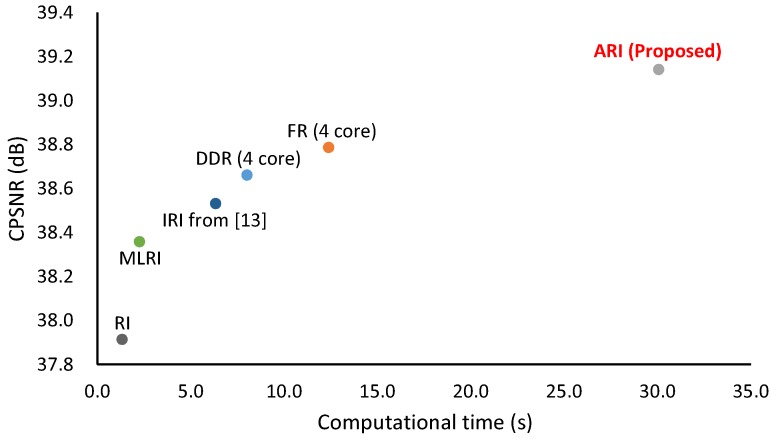
Computational time versus CPSNR performance plot of state-of-the-art algorithms, in which IMAX and Kodak 30 images are used. The computational time is averaged for the 30 images. Note that the computational time of IRI was extracted from [12] because IRI was not executable in our computation environment. DDR and FR exploit parallelized implementation (four cores in our environment).

Figure 11 shows the computational time versus CPSNR performance plot of state-of-the-art algorithms, in which the average computational time of the IMAX and Kodak 30 images is measured using a desktop PC (Intel Xeon CPU E5-1603 v3 2.80-GHz processor with 80-GB RAM). Note that the computational time of IRI was extracted from [12] because the authors only provide result images, and we cannot run IRI in our computation environment. The other algorithms were executed using MATLAB R2016b. DDR and FR exploit parallelized implementation (four cores in our environment). One can see that the computational time and the CPSNR performance are positively correlated. IRI takes more time than RI because IRI iterates RI. ARI takes more time than IRI because ARI iterates both RI and MLRI and combines them. The computational time of DDR and FR is between that of IRI and that of ARI. Because each directional interpolation in Figure 4 can be parallelized, our future work is to reduce the computation time of ARI with parallelized implementation.

### 6.2. Performance of Multispectral Demosaicking Algorithms

To evaluate the performance of multispectral demosaicking algorithms for the five-band MSFA of Figure 1c, we conducted the same comparisons as performed in [58] and [7].

The first comparison was performed on the five-band image dataset used in [58]. The dataset contains 16 scenes of size 1824 × 1368 pixels. The five-band images were mosaicked according to the five-band MSFA of Figure 1c and demosaicked using compared demosaicking algorithms. Our proposed ARI (Code available at http://www.ok.sc.e.titech.ac.jp/res/MSI/MSIdata.html.) was compared with four existing algorithms, BTES [55], AKU [56], GF [57] and RI [58]. The RI algorithm is one of the current state-of-the-art algorithms. We evaluated the PSNR performance of the five-band images and the PSNR and the CIEDE2000 [70] performance of the standard RGB (sRGB) images. The sRGB images were generated from the five-band images using a calibrated color transformation matrix from the five bands to the sRGB.

**Figure 12:**
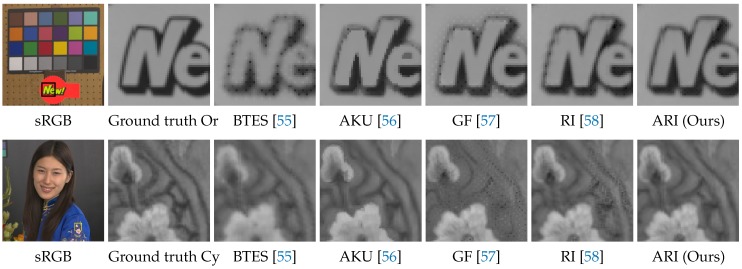
Visual comparisons of the demosaicking results on the five-band image dataset.

Table 3 shows the average PSNR and CIEDE2000 performance of all 16 scenes. One can see that ARI outperforms all existing algorithms in the numerical evaluation. Figure 12 shows visual comparisons of the demosaicking results for the Or and the Cy band images. One can see that ARI can sharply generate the images without the severe zipper artifacts and blurring that appear in the results of the existing algorithms.

The second comparison was performed on three 31-band multispectral image datasets, the CAVE [66], the TokyoTech [7] and the NUS [71] datasets. The CAVE and the TokyoTech datasets were captured using a monochrome camera with a liquid crystal tunable filter [72]. The NUS dataset was captured using a Specim’s hyperspectral camera (http://www.specim.fi/products/pfd-65-v10e/). The CAVE dataset contains 32 scenes of size 512 × 512 pixels. The TokyoTech dataset contains 30 scenes of size 500 × 500 pixels. The NUS dataset contains 66 scenes (training 41 scenes and testing 25 scenes for the purpose of [71]) of different pixel resolutions. In the NUS dataset, we used the testing 25 scenes for evaluation.

**Figure 13:**
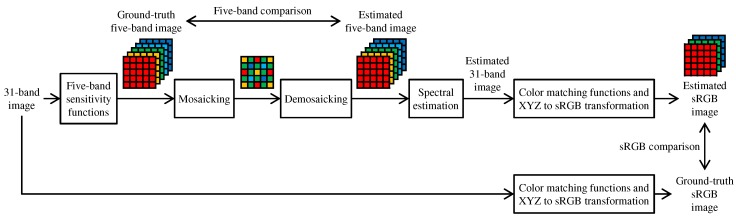
Flow of experimental comparison using 31-band multispectral datasets.

Figure 13 shows the flow of experimental comparison using the 31-band datasets. As shown in the bottom row of Figure 13, ground truth sRGB images were simulated from the 31-band images based on the XYZ color matching functions and the XYZ-to-sRGB transformation matrix with a correct white point. As shown in the top row of Figure 13, ground truth five-band images were simulated from the 31-band images using the spectral sensitivity of the five-band MSFA as described in [7]. To generate the five-band images, we used the CIE D65 illumination for the CAVE and the TokyoTech datasets. For the NUS dataset, we used given illumination spectrum for each scene. Then, the ground truth five-band images were mosaicked according to the five-band MSFA of Figure 1c and demosaicked using compared demosaicking algorithms. Finally, the demosaicked five-band images were converted to sRGB images using a linear model-based spectral reflectance (31-band image) estimation [73] and a rendering process from the spectrum to the sRGB with the XYZ color matching functions. The ground truth and the estimated images were compared in the five-band and the sRGB domains. Our proposed ARI was compared with three existing algorithms, BTES [55], GF [7] and RI [58]. We evaluated the PSNR performance of the five-band images and PSNR, CPSNR, DeltaE (Euclidean distance in the CIE Lab space) and CIEDE2000 performance of the sRGB images.

**Figure 14:**
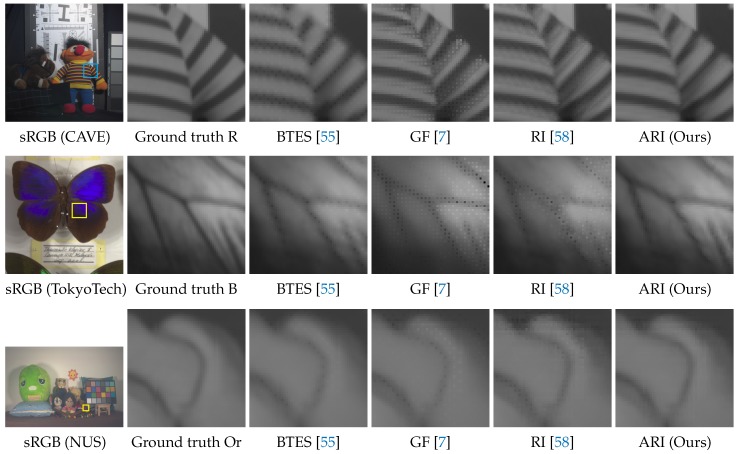
Visual comparisons of the demosaicking results on CAVE, TokyoTech and NUS datasets.

Table 4 presents a summary of numerical performance. One can see that ARI generally outperforms the existing algorithms and yields results with lower colorimetric errors. Figure 14 shows visual comparisons of the demosaicking results for the R band image of the CAVE dataset, the B band image of the TokyoTech dataset and the Or band image of the NUS dataset. One can see that ARI can significantly reduce the zipper artifacts that are apparent in the results of the existing algorithms. Both the numerical and visual comparisons validate that our proposed ARI can achieve state-of-the-art performance for the task of multispectral image demosaicking. As demonstrated in the above results, our proposed ARI is very effective when (i) one of spectral channels has a higher sampling density and (ii) each spectral channel is regularly sampled in horizontal/vertical directions or diagonal directions.

## 7. Conclusions

In this paper, we proposed a novel algorithm for both color and multispectral image demosaicking. Our proposed algorithm is based on a new interpolation technique called ARI that improves existing RI-based algorithms by the adaptive combination of two RI-based algorithms and the adaptive selection of a suitable iteration number at each pixel. Experimental comparisons using standard color image datasets demonstrated that ARI can improve existing RI-based algorithms by approximately 0.6 dB in CPSNR performance and can outperform state-of-the-art algorithms based on training images. Experimental comparisons using multispectral image datasets demonstrated that ARI can achieve state-of-the-art performance also for the task of multispectral image demosaicking.

## Figures and Tables

**Table 1:** Average PSNR and CPSNR performance for the standard IMAX and Kodak datasets. The bold typeface represents the best performance.

Algorithm	IMAX				Kodak				IMAX + Kodak			
	R	G	B	CPSNR	R	G	B	CPSNR	R	G	B	CPSNR
AP [49]	32.91	35.15	32.37	33.27	39.99	43.24	39.69	40.69	35.74	38.39	35.30	36.24
PCSD [26]	34.61	38.10	33.44	34.90	39.61	41.62	39.13	39.98	36.61	39.51	35.72	36.94
SA [25]	32.73	34.73	32.10	32.98	39.99	43.37	39.55	40.65	35.63	38.18	35.08	36.05
AHD [27]	33.00	36.97	32.16	33.49	38.81	40.84	38.42	39.22	35.32	38.52	34.66	35.78
DLMMSE [28]	34.03	37.99	33.04	34.47	41.17	43.94	40.51	41.62	36.89	40.37	36.03	37.33
FD [45]	33.12	35.86	32.56	33.58	40.57	44.10	39.98	41.20	36.10	39.15	35.53	36.63
HEID [36]	31.63	34.40	31.26	32.16	40.44	43.73	39.88	41.02	35.16	38.13	34.71	35.70
VCD [29]	34.15	37.18	33.43	34.58	40.93	43.92	40.45	41.51	36.86	39.88	36.24	37.35
LPA [32]	34.36	37.88	33.30	34.72	41.66	44.46	41.00	42.12	37.28	40.51	36.38	37.68
DFPD [30]	33.80	37.21	33.00	34.27	40.26	42.54	39.86	40.72	36.39	39.34	35.74	36.85
WA [51]	33.25	36.81	32.61	33.82	40.68	43.41	40.29	41.24	36.22	39.45	35.68	36.79
HPHD [31]	35.33	39.39	34.30	35.73	40.55	42.28	39.97	40.82	37.42	40.55	36.57	37.77
SSD [39]	35.02	38.27	33.80	35.23	38.83	40.51	39.08	39.40	36.54	39.17	35.91	36.90
RAD [52]	33.46	37.15	33.28	34.26	39.74	43.81	40.06	40.83	35.97	39.82	35.99	36.89
LSSC [16]	36.03	38.84	34.73	36.17	**42.42**	**45.79**	**41.64**	**42.93**	38.59	41.62	37.49	38.87
GBTF [34]	33.98	37.34	33.07	34.38	41.74	44.84	41.04	42.23	37.09	40.34	36.25	37.52
OAP [50]	32.94	35.16	32.31	33.26	40.13	43.26	39.78	40.79	35.81	38.40	35.30	36.27
IGD [33]	34.33	37.38	33.46	34.70	41.72	44.85	41.10	42.26	37.29	40.37	36.51	37.72
NAT [40]	36.31	39.82	34.50	36.27	38.35	40.50	37.95	38.79	37.13	40.09	35.88	37.28
CS [43]	35.56	38.84	34.58	35.92	41.01	44.17	40.12	41.43	37.74	40.97	36.80	38.12
ESF [37]	33.45	36.36	32.67	33.83	41.48	44.81	40.84	42.04	36.66	39.74	35.94	37.11
MSG [35]	34.38	37.65	33.39	34.72	42.14	45.31	41.40	42.63	37.48	40.72	36.59	37.89
RI [8]	36.11	39.99	35.38	36.50	39.72	42.17	38.88	40.03	37.55	40.86	36.78	37.91
AICC [42]	35.41	39.11	34.00	35.60	41.52	44.60	41.03	42.09	37.86	41.30	36.81	38.20
ECC [13]	36.69	39.99	35.32	36.79	39.94	42.17	39.01	40.17	37.99	40.86	36.80	38.14
MLRI [10]	36.72	40.23	35.59	36.92	40.24	42.31	39.51	40.52	38.13	41.06	37.16	38.36
IRI [11]	36.62	40.28	35.79	36.98	40.27	43.48	39.72	40.85	38.08	41.56	37.36	38.53
DDR [15]	37.09	40.33	35.62	37.15	40.71	42.63	39.89	40.92	38.54	41.25	37.33	38.66
FR [15]	**37.48**	**41.00**	35.81	37.47	40.55	42.40	39.75	40.75	38.70	41.56	37.38	38.79
ARI (Previous)	37.37	40.68	36.05	37.49	40.87	43.75	40.25	41.37	38.77	**41.91**	37.73	39.04
ARI (Ours)	37.45	40.68	**36.21**	**37.60**	41.06	43.75	40.32	41.47	**38.90**	**41.91**	**37.86**	**39.14**

**Table 2:** Average structural similarity (SSIM) performance for the standard IMAX and Kodak datasets. The bold typeface represents the best performance.

Algorithm	IMAX				Kodak				IMAX+Kodak			
	R	G	B	Ave.	R	G	B	Ave.	R	G	B	Ave.
AP [49]	0.9237	0.9420	0.8860	0.9172	0.9845	0.9906	0.9822	0.9857	0.9480	0.9614	0.9245	0.9446
PCSD [26]	0.9397	0.9649	0.9040	0.9362	0.9782	0.9844	0.9753	0.9793	0.9551	0.9727	0.9325	0.9535
SA [25]	0.9200	0.9346	0.8780	0.9109	0.9844	0.9909	0.9816	0.9856	0.9458	0.9571	0.9195	0.9408
AHD [27]	0.9268	0.9614	0.8836	0.9239	0.9786	0.9854	0.9750	0.9796	0.9475	0.9710	0.9201	0.9462
DLMMSE [28]	0.9339	0.9647	0.8944	0.9310	0.9853	0.9913	0.9822	0.9863	0.9545	0.9753	0.9295	0.9531
FD [45]	0.9248	0.9481	0.8868	0.9199	0.9840	0.9919	0.9811	0.9856	0.9485	0.9656	0.9245	0.9462
HEID [36]	0.9043	0.9294	0.8615	0.8984	0.9855	0.9914	0.9827	0.9865	0.9368	0.9542	0.9100	0.9336
VCD [29]	0.9335	0.9570	0.9017	0.9307	0.9798	0.9864	0.9786	0.9816	0.9520	0.9688	0.9325	0.9511
LPA [32]	0.9402	0.9652	0.9021	0.9358	0.9876	0.9924	0.9848	0.9883	0.9592	0.9760	0.9352	0.9568
DFPD [30]	0.9352	0.9618	0.8983	0.9318	0.9842	0.9893	0.9816	0.9850	0.9548	0.9728	0.9316	0.9531
WA [51]	0.9272	0.9558	0.8875	0.9235	0.9858	0.9913	0.9835	0.9869	0.9506	0.9700	0.9259	0.9489
HPHD [31]	0.9488	0.9736	0.9188	0.9471	0.9822	0.9870	0.9790	0.9827	0.9621	0.9790	0.9429	0.9613
SSD [39]	0.9509	0.9730	0.9169	0.9469	0.9767	0.9826	0.9755	0.9783	0.9612	0.9768	0.9403	0.9595
RAD [52]	0.9288	0.9588	0.8997	0.9291	0.9834	0.9915	0.9825	0.9858	0.9506	0.9719	0.9328	0.9518
LSSC [16]	0.9555	0.9737	0.9278	0.9523	**0.9882**	**0.9934**	0.9845	0.9887	0.9686	0.9816	0.9504	0.9669
GBTF [34]	0.9370	0.9618	0.8986	0.9325	0.9876	0.9927	0.9848	0.9884	0.9572	0.9742	0.9331	0.9548
OAP [50]	0.9225	0.9418	0.8837	0.9160	0.9845	0.9906	0.9823	0.9858	0.9473	0.9614	0.9231	0.9439
IGD [33]	0.9406	0.9623	0.9054	0.9361	0.9874	0.9926	0.9847	0.9882	0.9594	0.9744	0.9371	0.9570
NAT [40]	0.9560	0.9749	0.9219	0.9510	0.9769	0.9841	0.9726	0.9779	0.9643	0.9786	0.9422	0.9617
CS [43]	0.9563	0.9755	0.9306	0.9541	0.9858	0.9915	0.9821	0.9864	0.9681	0.9819	0.9512	0.9671
ESF [37]	0.9326	0.9570	0.8935	0.9277	0.9869	0.9924	0.9843	0.9879	0.9543	0.9712	0.9298	0.9517
MSG [35]	0.9395	0.9630	0.9027	0.9351	**0.9882**	0.9931	**0.9855**	**0.9889**	0.9590	0.9751	0.9358	0.9566
RI [8]	0.9597	0.9797	0.9404	0.9599	0.9822	0.9886	0.9770	0.9826	0.9687	0.9833	0.9550	0.9690
AICC [42]	0.9586	0.9763	0.9288	0.9546	0.9866	0.9920	0.9841	0.9876	0.9698	0.9826	0.9509	0.9678
ECC [13]	0.9639	0.9797	0.9417	0.9618	0.9822	0.9886	0.9766	0.9825	0.9713	0.9833	0.9557	0.9701
MLRI [10]	0.9640	0.9811	0.9420	0.9624	0.9843	0.9888	0.9801	0.9844	0.9721	0.9842	0.9572	0.9712
IRI [11]	0.9634	0.9816	0.9430	0.9627	0.9832	0.9902	0.9791	0.9842	0.9713	0.9850	0.9575	0.9713
DDR [15]	0.9566	0.9752	0.9340	0.9553	0.9794	0.9831	0.9758	0.9794	0.9657	0.9784	0.9507	0.9649
FR [15]	0.9588	0.9768	0.9360	0.9572	0.9785	0.9823	0.9747	0.9785	0.9667	0.9790	0.9515	0.9657
ARI (Previous)	0.9666	**0.9828**	0.9434	0.9643	0.9832	0.9895	0.9791	0.9840	0.9733	**0.9855**	0.9577	0.9721
ARI (Ours)	**0.9679**	**0.9828**	**0.9465**	**0.9657**	0.9831	0.9895	0.9795	0.9840	**0.9740**	**0.9855**	**0.9597**	**0.9730**

**Table 3:** Average PSNR and CIEDE2000 [70] performance of all 16 scenes in the five-band dataset. The bold typeface represents the best performance.

Algorithm	PSNR								CIEDE2000
	R	Or	G	Cy	B	s R	sG	sB	
BTES [55]	49.38	45.00	48.60	42.78	44.93	34.46	42.95	36.36	2.91
AKU [56]	52.19	47.80	48.78	45.38	48.06	38.14	44.20	39.53	2.34
GF [57]	53.12	51.06	49.61	47.94	48.89	40.75	45.73	40.51	2.06
RI [58]	54.93	52.31	51.08	49.42	49.86	42.49	47.19	41.26	1.88
ARI (Ours)	**55.45**	**52.81**	**51.63**	**49.82**	**50.15**	**43.00**	**47.74**	**41.52**	**1.79**

**Table 4:** Average PSNR, CPSNR, DeltaE and CIEDE2000 [70] performance on the CAVE, TokyoTech and NUS datasets. The bold typeface represents the best performance.

Light	Dataset	Algorithm	PSNR R	PSNR Or	PSNR G	PSNR Cy	PSNR B	5band PSNR Ave.	sRGB PSNR Ave.	CPSNR	DeltaE	CIEDE 2000
D65	CAVE	BTES [55]	42.60	39.41	46.54	37.83	40.46	41.37	36.94	35.81	2.85	3.81
		GF [7]	45.36	44.76	48.06	44.68	43.96	45.36	40.00	39.38	2.35	3.09
		RI [58]	46.21	46.22	**49.77**	**45.84**	45.92	46.79	40.76	40.07	2.38	3.19
		ARI (Ours)	**47.04**	**46.65**	49.66	45.82	**46.48**	**47.13**	**40.84**	**40.18**	**2.33**	**3.12**
D65	TokyoTech	BTES [55]	38.27	37.05	45.51	36.34	39.11	39.26	35.58	34.28	2.07	2.58
		GF [7]	43.82	43.28	46.57	42.92	43.62	44.04	39.00	38.07	1.56	1.88
		RI [58]	44.59	44.96	48.23	44.39	44.90	45.41	40.08	39.03	1.46	1.74
		ARI (Ours)	**45.65**	**45.82**	**48.84**	**45.16**	**46.05**	**46.30**	**40.63**	**39.63**	**1.36**	**1.63**
Given	NUS	BTES [55]	49.36	46.41	58.06	51.72	55.81	52.27	39.10	36.39	1.73	2.87
		GF [7]	52.36	51.46	56.90	57.01	56.51	54.85	40.11	37.40	1.68	2.78
		RI [58]	53.77	53.22	59.35	57.96	57.98	56.46	40.47	37.72	1.65	2.73
		ARI (Ours)	**54.79**	**54.07**	**59.52**	**58.36**	**58.60**	**57.07**	**40.57**	**37.82**	**1.63**	**2.71**
